# Mannose controls mesoderm specification and symmetry breaking in mouse gastruloids

**DOI:** 10.1016/j.devcel.2024.03.031

**Published:** 2024-04-17

**Authors:** Chaitanya Dingare, Dominica Cao, Jenny Jingni Yang, Berna Sozen, Benjamin Steventon

**Affiliations:** 1 Deptartment of Genetics, University of Cambridge, Downing Site, Cambridge CB2 3EH, UK; 2 Department of Genetics, Yale School of Medicine, Yale University, New Haven, CT, USA; 3 Yale Stem Cell Centre, Yale University, New Haven, CT, USA; 4 Department of Obstetrics, Gynaecology and Reproductive Sciences, Yale School of Medicine, Yale University, New Haven, CT, USA

## Abstract

Patterning and growth are fundamental features of embryonic development that must be tightly coordinated. To understand how metabolism impacts early mesoderm development, we used mouse embryonic stem-cell-derived gastruloids, that co-expressed glucose transporters with the mesodermal marker *T/Bra*. We found that the glucose mimic, 2-deoxy-D-glucose (2-DG), blocked *T/Bra* expression and abolished axial elongation in gastruloids. However, glucose removal did not phenocopy 2-DG treatment despite a decline in glycolytic intermediates. As 2-DG can also act as a competitive inhibitor of mannose in protein glycosylation, we added mannose together with 2-DG and found that it could rescue the mesoderm specification both *in vivo* and *in vitro*. We further showed that blocking production and intracellular recycling of mannose abrogated mesoderm specification. Proteomics analysis demonstrated that mannose reversed glycosylation of the Wnt pathway regulator, secreted frizzled receptor Frzb. Our study showed how mannose controls mesoderm specification in mouse gastruloids.

## Graphical abstract

**Graphical abstract:**
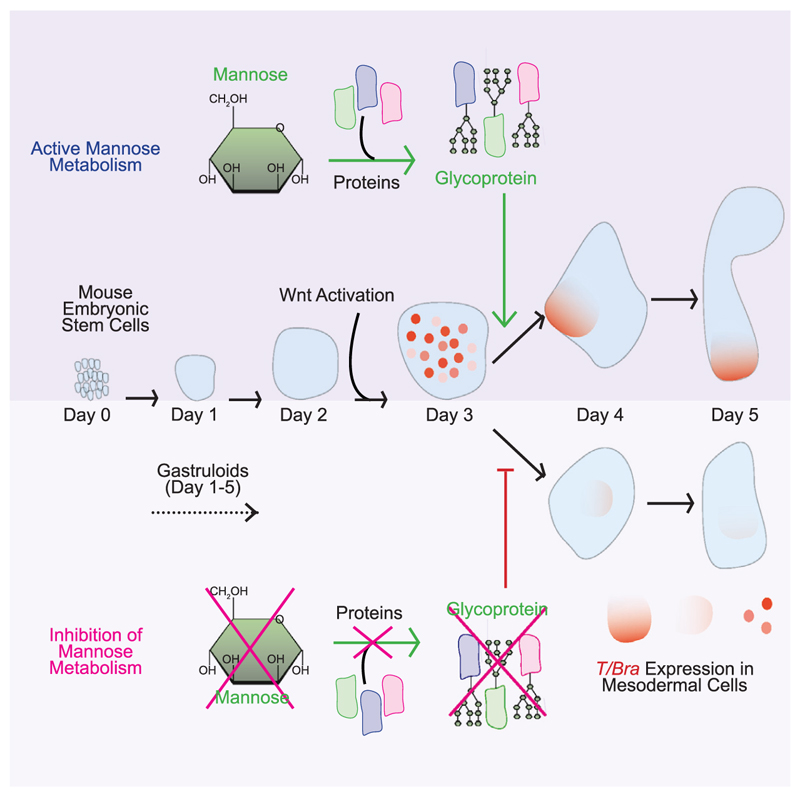

## Introduction

During embryonic development, cell differentiation must be coupled with cell growth and proliferation to yield well-proportioned tissues and organs. Pre-implantation mouse embryos rely on pyruvate as a carbon source.^1^ However, as gastrulation begins, peri- and post-implantation embryos rely more on glucose, concomitant with an acceleration in embryo size from 600 cells to 90,000 cells in just 2 days.^2–5^ This raises a key question of how glucose metabolism is coupled to cell fate specification to coordinate growth, patterning, and morphogenesis. Glucose metabolism is comprised a complex set of various metabolic pathways including various catabolic reactions and anabolic reactions. Additionally, glucose is converted into other hexoses such as mannose, fucose, and galactose. These hexoses are further modified to form metabolic intermediates such as N-acetylglucosamine and N-acetyl galactosamine that are used in protein glycosylation, an important post-translational modification (PTM) that dictates the intracellular localization of proteins. Therefore, there are multiple ways in which glucose metabolism has the potential to coordinate growth and cell fate specification.^6^

Qualitative studies on chicken embryos have shown a requirement for glucose in the tailbud of the embryo, highlighting the importance of glucose metabolism in this tissue.^7^ This finding was recently corroborated by blocking glucose metabolism using a glycolysis inhibitor, 2-deoxy-D-glucose (2-DG) and revealed an inhibition of mesoderm specification via inhibition of the Wnt signaling pathway.^8^ It was later shown that glycolysis-dependent lactic acid produced by the tailbud cell population was responsible for maintaining a basic intracellular pH subsequently regulating the acetylation of β-catenin and activation of Wnt signaling.^8,9^ In addition, tailbud explants from the mouse embryo have disrupted pre-somitic mesoderm (PSM) development and somitogenesis when incubated in the glucose free medium. It was subsequently demonstrated that glycolytic flux also regulates Wnt signaling and the PSM development in the mouse embryo.^10,11^ Together, these studies highlight a conserved importance of glucose metabolism in mesoderm development during tailbud stages of development. It is likely that glucose metabolism is playing an important role in gastrulation stages also, as mouse mutants for glucose transporters Glut1 and Glut3 as well as glycolytic enzymes hexokinase II and glucose phosphate isomerase show defects in germ layer specification and die post gastrulation.^4,12–14^ However, a minimal system is required to isolate specific mechanisms that couple metabolism and developmental signaling in embryonic cells.

Gastruloids are aggregates derived from mouse embryonic stem cells (ESCs).^15^ *Brachyury* (*T/Bra*)-expressing mesodermal cells are specified upon Wnt pathway activation and subsequently aggregate at one pole marking it as the posterior, breaking symmetry of the gastruloids.^15,16^ These specification, patterning, and morphogenetic events resemble those *in vivo* mouse embryos, yet occur in the absence of extra-embryonic tissues and signals.^16,17^ Furthermore, in gastruloids, mesoderm specification and axial elongation are temporally separated, making it possible to perturb metabolic enzymes in time-windows ascribed to each biological process using small molecule inhibitors. The effect of such treatments can be easily monitored in a high-throughput and non-invasive manner. Therefore, gastruloids offer a simple and high-throughput model system to shed light on the role of glucose metabolism in mesoderm specification.^18^

To address the role of glucose metabolism in mesoderm specification, we first analyzed the expression pattern of glucose transporters and found that *Slc2a1* and *Slc2a3* are specifically expressed in the mesodermal cells and neural cells. 2-DG treatment specifically blocked the specification of mesodermal cells, impairing axial elongation. Even though our metabolomics data suggested that 2-DG efficiently blocked glycolysis in the gastruloids, removing glucose completely from the medium did not affect mesoderm specification and axial elongation. These results highlighted a glycolysis-independent effect of 2-DG on mesoderm specification. 2-DG shares a structural similarity with D-Mannose it can become incorporated in place of D-Mannose during glycosylation of different proteins.^19^ Therefore, we tested the possibility that the 2-DG phenotype resulted from competitive inhibition of mannose utilization by adding D-Mannose to 2-DG-treated gastruloids, which rescued mesoderm specification and gastruloid elongation. We corroborated these findings in mouse embryos where supplementing with mannose rescued the defects in mesoderm specification and proximo-distal elongation of the primitive streak caused by 2-DG. Finally, we tested the requirement for mannose production during development and assessed alterations in the glycoproteome following 2-DG and mannose treatments. Our study demonstrated a role for mannose in mesoderm specification during gastrulation.

## Results

### 2-DG, a competitive inhibitor of hexokinase, inhibited mesoderm specification and gastruloid elongation

Cellular glucose uptake is facilitated by a special type of transporter belonging to the solute carrier family 2 (Slc).^20^ To test whether gastruloids expressed glucose transporters, we analyzed publicly available single-cell RNA sequencing and tomo-seq databases.^21^ We found two genes encoding glucose transporters (*Slc2a1* and *Slc2a3*) that were expressed in the posterior region of the gastruloids, which harbors mesodermal and neural progenitor cell populations. We next performed hybridization chain reaction (HCR) staining for *Slc2a1* and *Slc2a3* mRNA along with mesodermal marker *T/Bra* and neural marker *Sox2*.^22^ We corroborated the mRNA expression analysis with immunostainings for Glut1 and Glut3 encoded by *Slc2a1* and *Slc2a3*, respectively. We found that the expression of both the transporters was gradually restricted to *T/Bra*- and *Sox2*-expressing cells between day 2 and day 5 (Figures S1A–S1F″). Both transporters were localized to the membrane of the cells with nuclear T/Bra localization (Figures S1G–S1N″).

**Figure 1:**
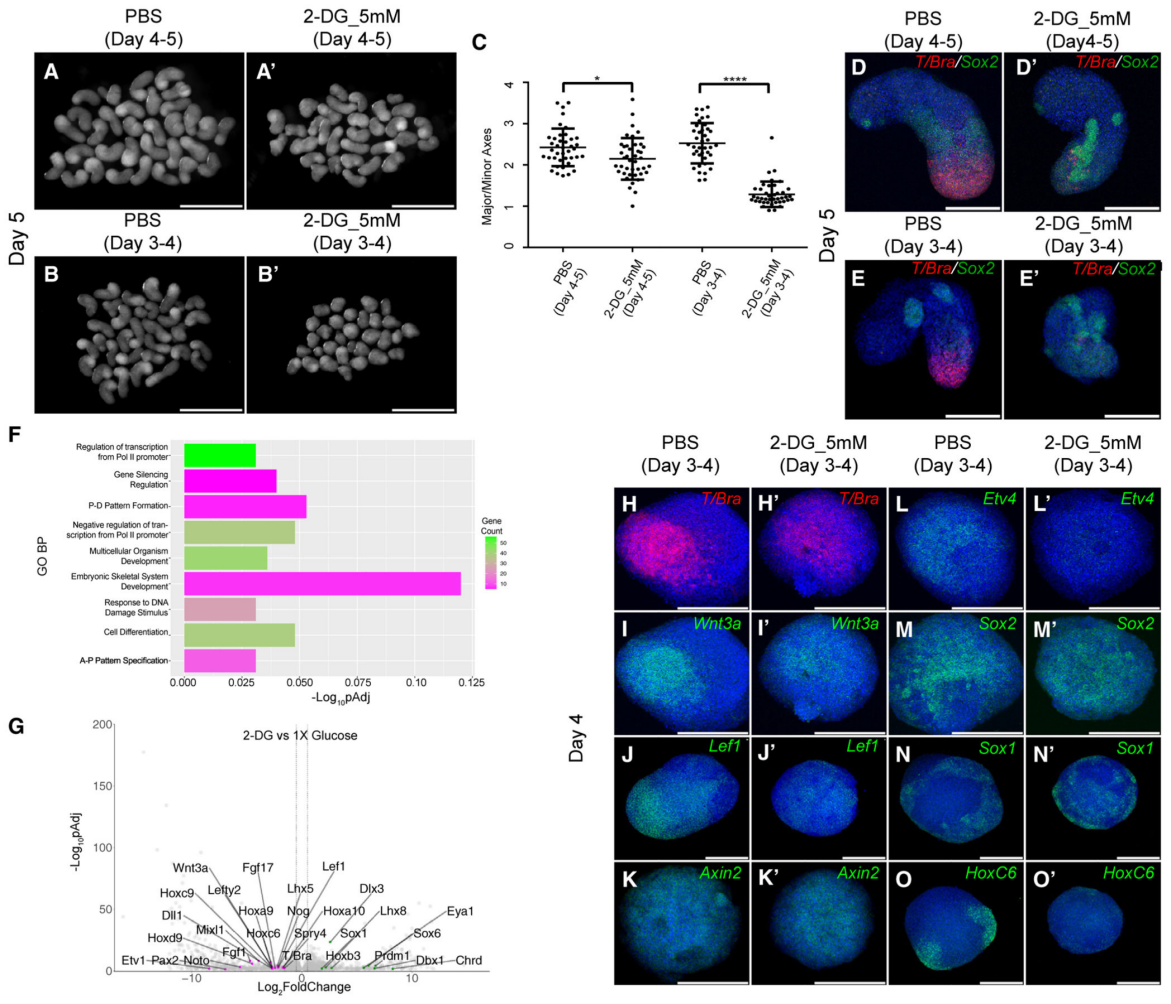
2-DG abrogated mesoderm specification (A and A′) Overview images showing day 5 gastruloids treated with PBS (A) and 2-DG (A′) between days 4 and 5 (*N* = 1). (B and B′) Overview images showing day 5 gastruloids treated with PBS (B) and 2-DG (B′) between days 3 and 4 (*N* > 3). (C) Graph showing the ratio of the major axis to minor axis of the gastruloids calculated from (A–B′) images (*N* = 1, PBS [days 4–5], *n* = 40; 2-DG [days 4–5], *n* = 41; PBS [days 3–4], *n* = 39; 2-DG [days 3–4], *n* = 41). *p* values for PBS (days 4–5) vs. 2-DG (days 4–5) = 0.0108 and for PBS (days 3–4) vs. 2-DG (days 3–4) < 0.0001. Data represented as mean ± SEM. (D–E′) Maximum intensity projections of HCR staining showing the expression pattern of *T/Bra* (red) and *Sox2* (green) in day 5 gastruloids treated with PBS (days 4–5) (D, *n* = 17), 2-DG (days 4–5) (D′, *n* = 17), PBS (days 3–4) (E, *n* = 19), and 2-DG (days 3–4) (E′, *n* = 18). (F) Bar graph showing Gene Ontology term analysis for biological processes (GO-BP) of the downregulated genes in 2-DG compared with PBS-treated gastruloids. FDR < 0.1. (G) Volcano plot showing the upregulated (green dots) and downregulated genes (magenta dots) in 2-DG-treated gastruloids. (cutoff for Log_2_FoldChange = −1.5, 1.5). (H–O′) Maximum intensity projections of HCR staining showing the expression patterns of *T/Bra* (H, *n* > 15), *Wnt3a* (I, *n* = 12), *Lef1* (J, *n* = 6), *Axin2* (K, *n* = 12), *Etv4* (L, *n* = 12), *Sox2* (M, *n* = 18), *Sox1* (N, n = 14), and *Hoxc6* (O, *n* = 10) in day 4 gastruloids treated with PBS (days 3–4) and *T/Bra* (H′, *n* > 15), *Wnt3a* (I′, *n* = 11), *Lef1* (J′, *n* = 6), *Axin2* (K′, *n* = 11), *Etv4* (L′, *n* = 12), *Sox2* (M′, *n* = 16), *Sox1* (N′, *n* = 12), and *Hoxc6* (O′, *n* = 10) in day 4 gastruloids treated with 2-DG (days 3–4). Scale bars, 2 mm for the overview images (A–B′) and 200 μm for the confocal images (D–E′ and H–O′). The blue staining denotes nuclei in all the confocal images.

To test whether blocking glycolysis affected gastruloid development, we treated them with 2-DG at defined intervals of the gastruloid protocol. When treated between days 4 and 5, i.e., during elongation, we observed a minor effect on gastruloid elongation (Figures 1A, 1A′, and 1C), together with a minor reduction in *T/Bra* expression relative to untreated controls (Figures 1D and 1D′). However, treatments prior to symmetry breaking and elongation (between days 3 and 4) led to an inhibition of elongation together with a loss of *T/Bra* expression (Figures 1B, B′, 1C, 1E, and 1E′). The expression of *Sox2* remained unchanged (Figures 1D, 1D′ 1E, and 1E′). Transcriptomic analysis of day 4 control and 2-DG-treated gastruloids revealed a differential expression of genes associated with anterior/posterior patterning, cell differentiation, and embryonic skeletal system development (Figure 1F). Many downregulated genes are related to mesoderm cell fate specification (*T/Bra, Wnt3a, Noto*, etc.) while the upregulated genes in 2-DG treated gastruloids were related to neural development (*Sox1, Dbx1*, and *Eya1*). Genes related to Wnt signaling (*Lef1*) and to fibroblast growth factor (FGF) signaling (*Spry4, Etv1*) were downregulated in 2-DG treated gastruloids. In addition, many 5′ (posteriorly expressed) Hox genes were shown to have reduced expression pointing toward the overall suppression of posterior fate specifications in the gastruloids upon 2-DG treatment (Figure 1G). Key expression changes were validated by HCR, i.e., *T/Bra* (Figures 1H and 1H′), *Wnt3a* (Figures 1I and 1I′), an FGF target gene *Etv4* (Figures 1L and 1L′), a Wnt target gene *Lef1* (Figures 1J and 1J′), and the late Hox gene *Hoxc6* (Figures 1O and 1O′) all showed a reduction in the 2-DG treated gastruloids. Expression of the neural marker *Sox2* (Figures 1M and 1M′) remained unchanged, and *Sox1* (Figures 1N and 1N′) showed a mild increase in the treated gastruloids. Although the *Wnt3a* mediated *T/Bra* expression is abrogated upon 2-DG treatment, we did not observe any effect on a broad range Wnt target *Axin2* (Figures 1K and 1K′). Altogether our transcriptomics and HCR data showed that 2-DG influenced mesoderm specification, posterior development, and signaling.

### Glucose deprivation did not impact mesoderm specification

**Figure 2:**
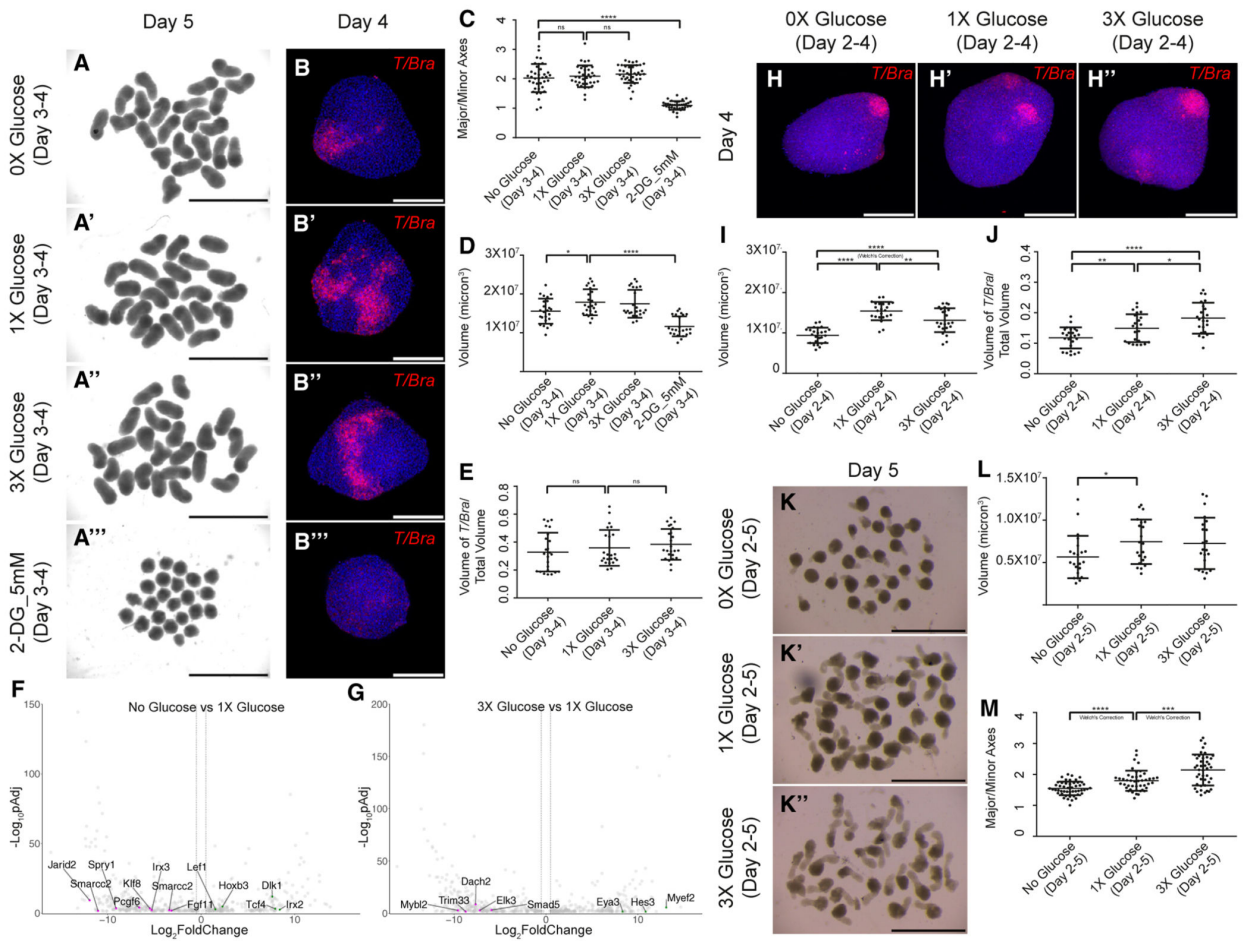
Complete removal of glucose did not affect mesoderm specification (A–A‴) Overview images showing day 5 gastruloids treated with 0× glucose (A), 1× glucose (A′), 3× glucose (A″), and 2-DG (A‴) between days 3 and 4 (*N* = 4). (B–B‴) Maximum intensity projections of the HCR staining showing the expression pattern of *T/Bra* in 0× glucose (B), 1× glucose (B′), 3× glucose (B″), and 2-DG (B‴)-treated gastruloids between days 3–4 (*N* = 3, *n* = 10 each experiment). (C) Graph showing the ratio of the major axis to minor axis of the gastruloids calculated from (A–A‴) images. (*N* = 2, 0× glucose *n* = 37, 1× glucose *n* = 36, 3× glucose *n* = 38, and 2-DG *n* = 37.) *p* values for 0× glucose vs. 1× glucose = 0.5286, for 1× glucose vs. 3× glucose = 0.3510, and for 0× glucose vs. 2-DG < 0.0001. Data represented as mean ± SEM. (D) Graph showing the volumes of the gastruloids treated with different concentrations of glucose and 2-DG between days 3–4 (0× glucose, 1× glucose, 3× glucose, and 2-DG, *N* = 3, *n* = 24). *p* values for 0× glucose vs. 1× glucose = 0.0194 and for 1× glucose vs. 2-DG < 0.0001. Data represented as mean ± SEM. (E) Graph showing the volume of the *T/Bra* expression domain normalized to the total volume of the gastruloids treated with different concentrations of glucose between days 3–4 (0×, 1×, and 3× glucose, *N* = 3, *n* = 24). *p* values for 0× vs. 1× glucose = 0.4332 and for 1× vs. 3× glucose = 0.4636. Data are represented as mean ± SEM. (F) Volcano plot showing the upregulated (green dots) and downregulated genes (magenta dots) in 0× glucose condition compared with 1× glucose. (cutoff for Log_2_FoldChange = −1.5, 1.5). (G) Volcano plot showing the upregulated (green dots) and downregulated genes (magenta dots) in 3× glucose condition compared with 1× glucose. (cutoff for Log_2_FoldChange = −1.5, 1.5). (H–H″) Maximum intensity projections of the HCR staining showing the expression pattern of *T/Bra* in 0× glucose (H, *N* = 3, *n* = 25), 1× glucose (H′, *N* = 3, *n* = 24), and 3× glucose (H″, *N* = 3, *n* = 24)-treated gastruloids. (I) Graph showing volumes of the gastruloids treated with different concentrations of glucose between days 2–4 (*N* = 3, 0× glucose *n* = 25, 1× glucose *n* = 23, and 3× glucose *n* = 24). *p* values for 0× glucose vs. 1× glucose < 0.0001, for 1× vs. 3× glucose = 0.0052, and for 0× vs. 3× glucose < 0.0001. Data are represented as mean ± SEM. (J) Graph showing the volume of *T/Bra* expression domain normalized to the total volume of the gastruloids treated with different concentrations of glucose between days 2–4 (*N* = 3, 0× glucose, n = 25; 1× glucose, *n* = 23; and 3× glucose, *n* = 23). *p* values for 0× glucose vs. 1× glucose = 0.0088, for 1× vs. 3× glucose = 0.0256, and for 0× vs. 3× glucose < 0.0001. Data represented as mean ± SEM. (K–K″) Overview images showing day 5 gastruloids treated with 0× glucose (A), 1× glucose (A′), and 3× glucose (A″) between days 2 and 5 (*N* = 3). (L) Graph showing volumes of the gastruloids treated with different concentrations of glucose between days 2–5 (*N* = 2, 0× glucose, *n* = 22; 1× glucose, *n* = 21; and 3× glucose *n* = 22). *p* values for 0× glucose vs. 1× glucose = 0.0262. Data are represented as mean ± SEM. (M) Graph showing the ratio of the major axis to minor axis of the gastruloids calculated from (K–K″) images. (*N* = 3, 0× glucose, *n* = 49; 1× glucose, *n* = 45; and 3× glucose, *n* = 43.) *p* values for 0× glucose vs. 1× glucose < 0.0001 and for 1× glucose vs. 3× glucose = 0.0002. Data are represented as mean ± SEM. Scale bars, 2 mm for the overview images (A–A‴ and K–K ″) and 200 μm for the confocal images (B–B‴ and H–H ″). The blue staining denotes nuclei in all the confocal images.

In addition to blocking glycolysis, 2-DG has been shown to have pleiotropic effects (e.g., induction of autophagy, apoptosis, interference with protein glycosylation causing endoplasmic reticulum (ER) stress^23^). Therefore, we next wanted to confirm whether the 2-DG mediated phenotype is linked to central glucose metabolism by removing glucose from the culture medium. In addition, we wanted to test whether higher glucose concentration could also impact mesoderm development. We incubated gastruloids in medium with no glucose (0×) or 3× higher glucose concentration between day 3 and 4 and transferred into 1× glucose medium until day 5, identical to the 2-DG treatment time window. Neither condition replicated the 2-DG phenotype, as revealed by the ratio of the major to the minor axes of gastruloids (Figures 2A–2A‴ and 2C), or an alteration of *T/Bra* expression (Figures 2B–2B‴). The overall volume of glucose-deprived gastruloids was reduced to a lesser extent than that of 2-DG treatment (Figure 2D), with the volume of the *T/Bra* expression scaling according to the total volume (Figure 2E). Transcriptomic analysis of 0×, 1×, and 3× glucose-treated gastruloids did not enrich any class of genes that are involved in developmental processes, mesoderm specification, or axial patterning, unlike 2-DG treatment (Figures 2F and 2G).

We next questioned whether a longer treatment with 0× or 3× glucose could affect mesoderm specification. To this end, we treated gastruloids with 0×, 1×, and 3× glucose concentrations from day 2 onward, i.e., coinciding with the mesodermal fate induction. Day 4 gastruloids treated with 0× or 3× glucose did not show any loss of *T/Bra* expression (Figures 2H–H″). Nevertheless, continuous glucose deprivation for 2 days significantly reduced the total volume of the gastruloids, with a similar reduction also observed in 3×-glucose-treated gastruloids (Figure 2I). Unlike the short-term treatments, the expression domain of *T/Bra* was not scaled in gastruloids treated with 0× or 3× glucose. In glucose-deprived gastruloids, the expression domain was significantly smaller while in 3× glucose-treated gastruloids it appeared mildly expanded (Figure 2J). We continued these different treatments until day 5 when the gastruloids elongated. Glucose-deprived gastruloids were much smaller as compared with both 1× and 3× glucose conditions (Figures 2K–2K″ and 2L). Gastruloids in all the three conditions elongated but at different lengths (Figure 2M). Overall, these results showed that 2-DG treatments have a stronger impact on mesoderm specification than glucose deprivation, suggesting that it might be acting independently of its glycolysis inhibitor function.

### 2-DG-treated gastruloids had a distinct metabolic signature as compared with glucose-deprived gastruloids

**Figure 3:**
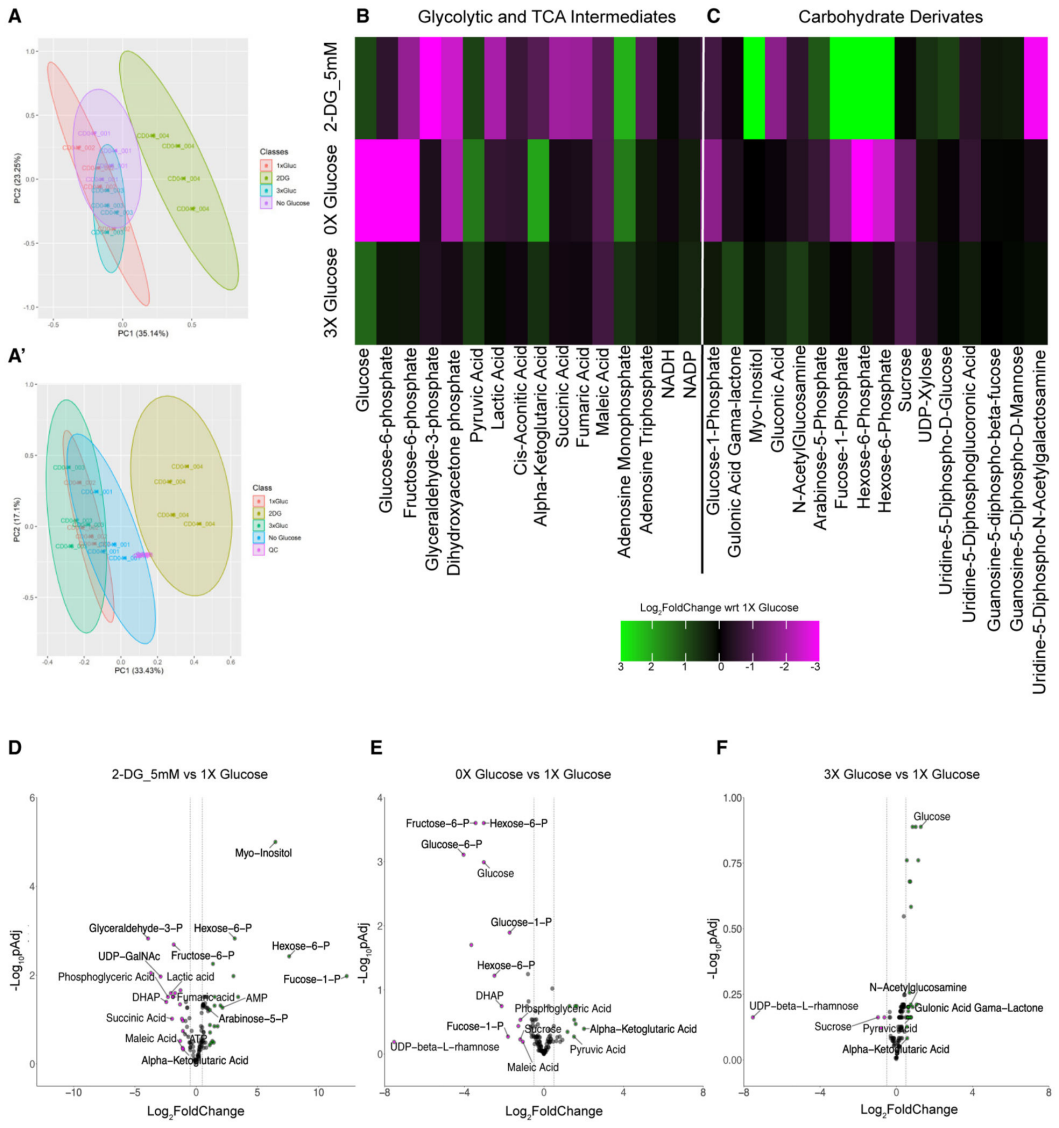
2-DG-treated gastruloids had a distinct metabolic signature compared with glucose-deprived gastruloids (A and A′) Variance scaled principal-component analysis of 4 classes (0× [001], 1× [002], 3× [003] glucose, and 2-DG_5 mM [004]) normalized to the total cell numbers in each class (A) and to signal of each metabolite normalized to the sum of all the signals within the sample (A′). The biological replicate numbers are denoted as CD41, CD42, CD43 and CD44. (B and C) Heatmap showing the Log_2_FoldChange in the level of glycolytic intermediates (B) and metabolites related to carbohydrate metabolism (C) in 0× glucose, 3× glucose, and 2-DG conditions, all compared with 1× glucose. (D–F) Volcano plot showing individual metabolites in lower abundance (magenta dots) and higher abundance (green dots) in 2-DG (D), 0× glucose (E), and 3× glucose (F), all compared with 1× glucose (cutoff for Log_2_FoldChange = −0.5, 0.5).

To better understand the discrepancy between 2-DG and no glucose conditions, we needed to understand how these treatments affected glucose metabolism and other related metabolic pathways. To this end, we performed untargeted metabolomics analysis of 2-DG, 0×, and 3× glucose conditions and compared this with 1× glucose. We first normalized the data by cell number to adjust metabolite levels to any differences in the sample size of the input material. A principal-component analysis (PCA) showed that 2-DG-treated samples are clearly distinct from the rest of the experimental conditions (Figure 3A). To further confirm that the observed difference in the 2-DG-treated gastruloid metabolome was not an artifact of our normalization method, we performed another PCA independent of the cell number where we normalized the mass spectrometry (MS) signal for each metabolite detected to the sum of all the MS signals detected in each condition (Figure 3A′). This analysis also showed 2-DG-treated samples to be separate from the rest of the experimental conditions highlighting their very distinct metabolic signature. 0×- and 3×-glucose-treated samples were still found to be closely related to the control (1× glucose) in the PCA plots (Figures 3A and 3A′).

At the pathway level, we found a reduction in the levels of glycolytic intermediates in the 2-DG-treated condition from the level of fructose-6-P, confirming that 2-DG indeed blocked the glycolytic pathway (Figure 3B). Notable intermediates reduced in level included fructose-6-P, glyceraldehyde-3-P, and importantly lactic acid (Figures 3B and 3D). The level of ATP was mildly reduced while that of AMP was moderately increased upon 2DG treatment (Figure 3B). TCA intermediates also showed an overall decrease in their levels (Figures 3B and 3D). However, unlike in 2-DG conditions, glucose deprivation drastically affected the glycolytic pathway from the level of glucose (Figure 3B). Importantly, glucose concentration was significantly lower than that in the control confirming the efficiency of the treatment (Figures 3B and 3E). Other intermediates that were depleted included glucose-6-P, fructose-6-P, and dihydroxyacetone phosphate (Figure 3E). Unlike in 2-DG, neither lactic acid nor TCA intermediates were impacted in 0× glucose condition (Figure 3B). In addition, ATP levels were not significantly different from that in the control while AMP levels were mildly increased (Figure 3B). One possible explanation for why TCA intermediates were not impacted by the absence of glucose is that cells might take up pyruvate directly from the media in which we were culturing all gastruloids. Consistent with this idea, we saw that pyruvate levels were mildly higher in the absence of glucose as compared with the control (Figures 3B and 3E).

Another important intermediate, which was significantly reduced in the 0× glucose condition but unchanged in 2-DG treatment, was glucose-1-P, an intermediate in the glycogen synthesis and the breakdown pathways (Figures 3B and 3E). These reduced levels could reflect either the slower production of glycogen or more rapid consumption to produce energy and other metabolic intermediates in the absence of glucose availability. As such, glucose-1-P could have been used to produce glucose-6-P to continue the glycolytic pathway in the 0× glucose condition.

From analyzing changes in specific metabolites, we observed an increase in glucose levels in the 3× glucose condition compared with 1× glucose confirming the efficiency of the treatment. However, none of the glycolytic and the TCA intermediates or any other sugar derivates showed any significant changes as compared with that in 1× glucose (Figures 3B and 3F). Nevertheless, amino acids were found to be more abundant in 3× glucose as compared with 1× glucose, hinting that excess glucose may be used in the amino acid biosynthetic pathway (Figure S2A). On the contrary, 2-DG and 0× glucose showed differences in the levels of glycolytic intermediates and sugar derivatives (Figures 3B and 3C). This metabolomics experiment helped us understand how different metabolic manipulations affected glycolysis both at the pathway and the individual metabolite levels.

Since some of the glycolytic intermediates are funneled into various other connected pathways, for example, to produce other hexoses in the cell, we observed an abundance of fucose-1-P in 2-DG, with a significant reduction in 0× glucose conditions (Figures 3C–3E). Fucose is produced from glucose and is an important sugar used in PTM in its activated form guanosine diphosphate (GDP)-fucose.^24^ GDP-fucose itself is produced from fucose-1-P in a reversible manner. Surprisingly, the levels of GDP-fucose were not different between 2-DG and 0× glucose treatments (Figure 3C). One explanation for this outcome in the 2-DG treated gastruloids was that fucose-1-P was also produced in the lysosome from the degradation of fucosylated proteins.^24^ A higher level of fucose-1-P, in 2-DG conditions could therefore be a product of the degradation of fucosylated proteins as 2-DG is known to induce ER stress, the unfolded protein response pathway and autophagy.^23^ In the case of no glucose conditions, the lack of glucose itself would be expected to lead to a lower level of fucose-1-P; therefore, we had to assume that remaining fucose-1-P has been used primarily to maintain the steady levels of GDP-fucose.

Our metabolomics data could detect other carbohydrate derivatives involved in glycosylation such as GDP-mannose, UDP-glucose, and N-acetylglucosamine (GlcNAc), but their levels were similar in both 2-DG and 0× glucose (Figure 3C). We could not detect the activated form of GlcNAc (UDP-GlcNAc), but the level of its epimer UDP-N-AcetylGalactosamine (UDP-GalNAc) was significantly reduced in 2-DG; however, not upon modulating glucose levels (Figures 3C and 3D).

Altogether, our metabolomics dataset revealed both similarities and differences between 2-DG and 0× glucose conditions with only 2-DG impacting intermediates involved in PTM.

### Mannose rescued 2-DG treatment phenotypes in mesodermal cells

**Figure 4:**
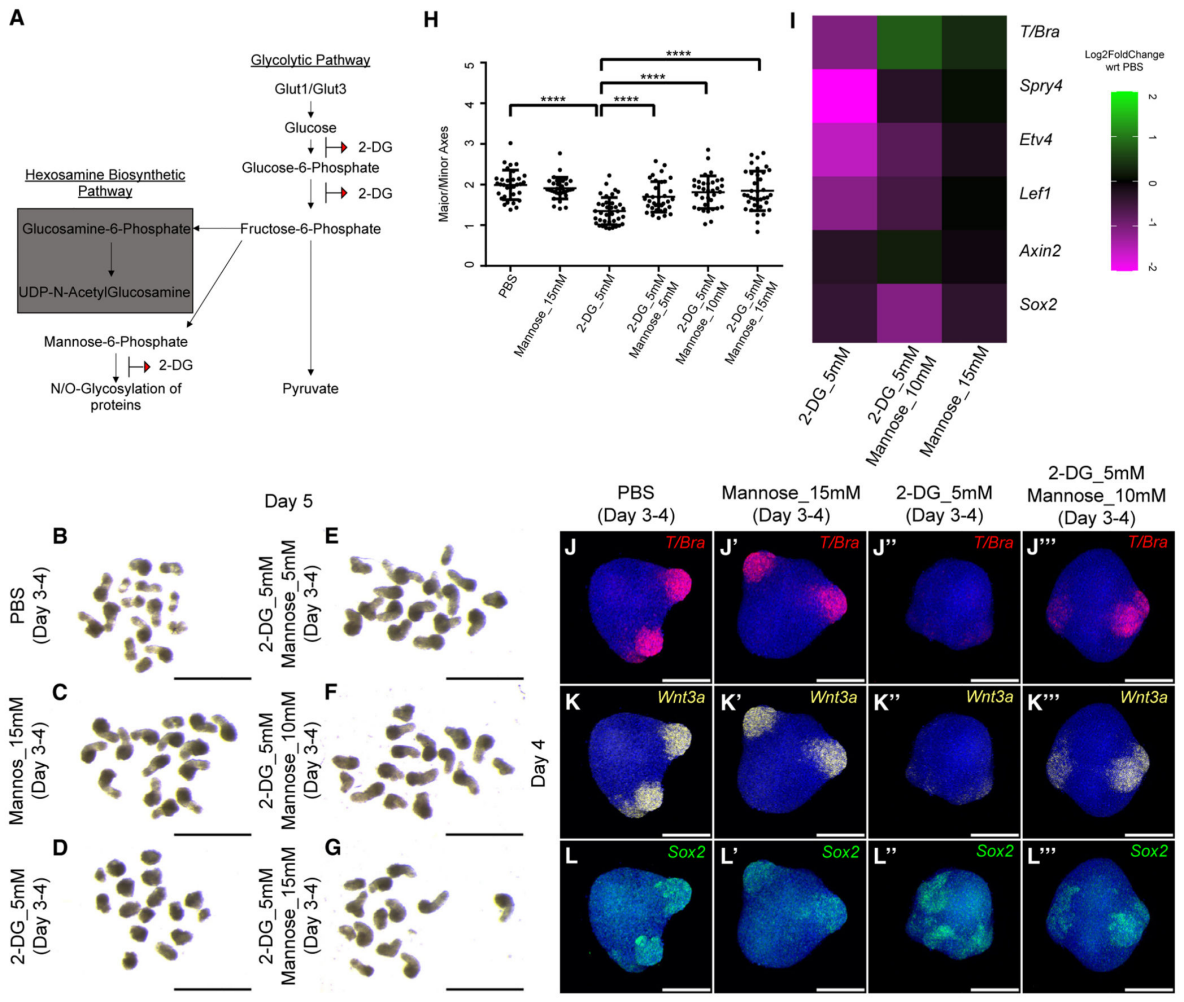
Mannose rescued the 2-DG-mediated phenotype (A) Schematic showing the glycolytic pathway and the targets of 2-DG. (B–G) Overview images showing day 5 gastruloids treated with PBS (B), Mannose_15 mM (C), 2-DG_5 mM (D), 2-DG_5 mM Mannose_5 mM (E), 2-DG_5 mM Mannose_10 mM (F), and 2-DG_5 mM Mannose_15 mM (G) between days 3–4 (*N* = 4). (H) Graph showing the ratio of the major axis to minor axis of the gastruloids calculated from (B–G) images (*N* = 3, PBS, *n* = 32; Mannose_15 mM, *n* = 33; 2-DG_5 mM, *n* = 37; 2-DG_5 mM Mannose_5 mM, *n* =35; 2-DG_5 mM Mannose_10 mM, *n* =34; 2-DG_5 mM Mannose_15 mM, *n* = 36). *p* values for PBS vs. 2-DG_5 mM < 0.0001, for 2-DG_5 mM vs. 2-DG_5 mM Mannose_5 mM < 0.0001, for 2-DG_5 mM vs. 2-DG_5 mM Mannose_10 mM < 0.0001, and for 2-DG_5 mM vs. 2-DG_5 mM Mannose_15 mM < 0.0001. Data are represented as mean ± SEM. (I) Heatmap showing the Log_2_FoldChange in the gene expression in 2-DG_5 mM, 2-DG_5 mM Mannose_10 mM and Mannose_15 mM compared with PBS-treated gastruloids (*N* = 2). (J–L‴) Maximum intensity projections of the HCR staining showing the expression patterns of *T/Bra* (J–J‴), *Wnt3a* (K–K‴), and *Sox2* (L–L‴) in PBS (J, K, and L: *N* = 2, *n* = 15); Mannose_15 mM (J′, K′, and L′: *N* = 2, *n* = 17); 2-DG_5 mM (J″, K″, and L″: *N* = 2, *n* = 19); and 2-DG_5 mM Mannose_10 mM (J‴, K‴, and L‴: *N* = 2, *n* = 20). Scale bars, 2 mm for the overview images (B–G) and 200 μm for the confocal images (J–L‴). The blue staining denotes nuclei in all the confocal images.

We next sought to determine the mechanism of 2-DG action in mesoderm specification, given that this was not phenocopied by glucose deprivation. 2-DG not only blocks the glycolytic pathway but also, due its structural similarity, competes with mannose and gets incorporated into the glycosyl chains of glycosylated proteins (Figure 4A). In a recent glycoproteomic study, clusters of proteins involved in germ layer and mesoderm specification were detected to be affected in an unbiased manner upon 2-DG treatment which otherwise were mannosylated.^19^ This prompted us to test whether 2-DG acted independently of the glycolytic pathway.

To determine whether mannose could rescue the phenotype by competing with 2-DG, we treated gastruloids with 5 mM of 2-DG and three different concentrations of mannose (5, 10, and 15 mM) between days 3 and 4. We then transferred them to medium containing no mannose and 2-DG until day 5. The overview images show that mannose alone did not affect gastruloid development as compared with the PBS control (Figures 4B and 4C). 2-DG treatment affected elongation when compared with both PBS and mannose treatments (compare Figure 4D with 4B and 4C), but when 2-DG was supplemented with mannose, the elongation was restored (compare Figure 4D with 4E–4G), similar to the PBS control (compare Figure 4B and 4C with 4E–4G). This rescue was also reflected in the aspect ratio the gastruloids (Figure 4H).

To confirm whether this elongation phenotype is mirrored by the restoration of mesodermal cell markers and signaling pathways, we performed qPCR analysis for *T/Bra*, FGF target genes *Etv4* and *Spry4*, the neural marker *Sox2*, and the general Wnt targets *Axin2* and *Lef1*. As shown before, the expression of *T/Bra* was downregulated in 2-DG as compared with the PBS control gastruloids (Figure 4I). However, upon addition of 10 mM mannose, the expression was restored to that in the control (Figure 4I). Alone, 15 mM mannose did not have any effect on *T/Bra* expression (Figure 4I). A similar trend was observed for an Fgf target gene *Spry4* (Figure 4I). Expression levels of *Etv4* and Wnt target gene *Lef1* were reduced in 2-DG, but mannose supplementation had a very little effect on the rescue of their expression levels (Figure 4I). Neither 2-DG, 2-DG with mannose nor mannose alone led to any changes in the expression of the neural gene *Sox2* and the broad range Wnt target gene *Axin2* (Figure 4I). We further confirmed the qPCR data with HCR. Expression of *T/Bra* and *Wnt3a* was lost upon 2-DG treatment (Figures 4J″–4K″) in comparison with that in PBS control (Figures 4J–4K). 15 mM mannose had no effect on the expression of both *Wnt3a* or *T/Bra* (Figures 4J′–4K′). However, the expression of both *Wnt3a* and *T/Bra* could be rescued by the addition of 10 mM mannose together with 2-DG (Figures 4J‴–4K‴). Expression of *Sox2* remained unchanged in all treatments (Figures 4L–4L‴). Altogether, our data showed that mannose addition was sufficient to restore the expression of mesodermal genes and the morphogenetic process of axial elongation in 2-DG-treated gastruloids.

To further confirm the importance of glycosylation in mesoderm specification, we treated gastruloids with 5-mM glucosamine. Although glucosamine is important in glycosylation, in excess, it interferes with the addition of mannose and glucose residues to the growing glycosyl chains.^25^ Similar to 2-DG treatment, glucosamine-treated gastruloids appeared less elongated as compared with their control counterparts (Figures S3A and S3A′). The ratio of the major to the minor axis was significantly reduced in glucosamine-treated gastruloids (Figure S3B). HCR staining for *T/Bra* revealed that indeed its expression was reduced in the glucosamine-treated gastruloids both in days 4 and 5 gastruloids (compare Figure S3E with S3G and S3F with S3H). However, unlike in 2-DG, the expression of *Wnt3a* was not completely diminished in day 4 gastruloids (compare Figure S3E′ with S3G′), in day 5 gastruloids, its expression was not localized to the posterior pole in the treated gastruloids as compared with the control (compare Figure S3F′ with S3H′). Expression of *Sox2* remained unchanged (compare Figure S3E″with S3G″ and S3F″ with S3H″). Altogether, our 2-DG and glucosamine dataset highlights the importance of mannose in protein glycosylation and subsequently mesoderm specification.

2-DG and glucosamine both affect N-glycosylation. To test whether O-glycosylation had any role to play in mesoderm specification, we treated gastruloids with ST045849, an inhibitor that blocks the activity of O-GlcNAc transferase. Day 5 gastruloids did not show any axial elongation phenotype as revealed by the aspect ratio measurements (Figures 3C, 3C′, and 3D). ST045849-treated gastruloids did not show any reduction in *T/Bra* or *Wnt3a* expression levels as compared with control both in day 4 and day 5 corroborating the lack of elongation phenotype (compare Figures S3I–S3I″ with S3K–S3K″ and Figures S3J–S3J″ with S3L–S3L″). Altogether these results showed that N-glycosylation is important in mesoderm specification.

### Mannose rescued 2-DG-mediated gastrulation phenotype *in vivo* mouse embryo

**Figure 5:**
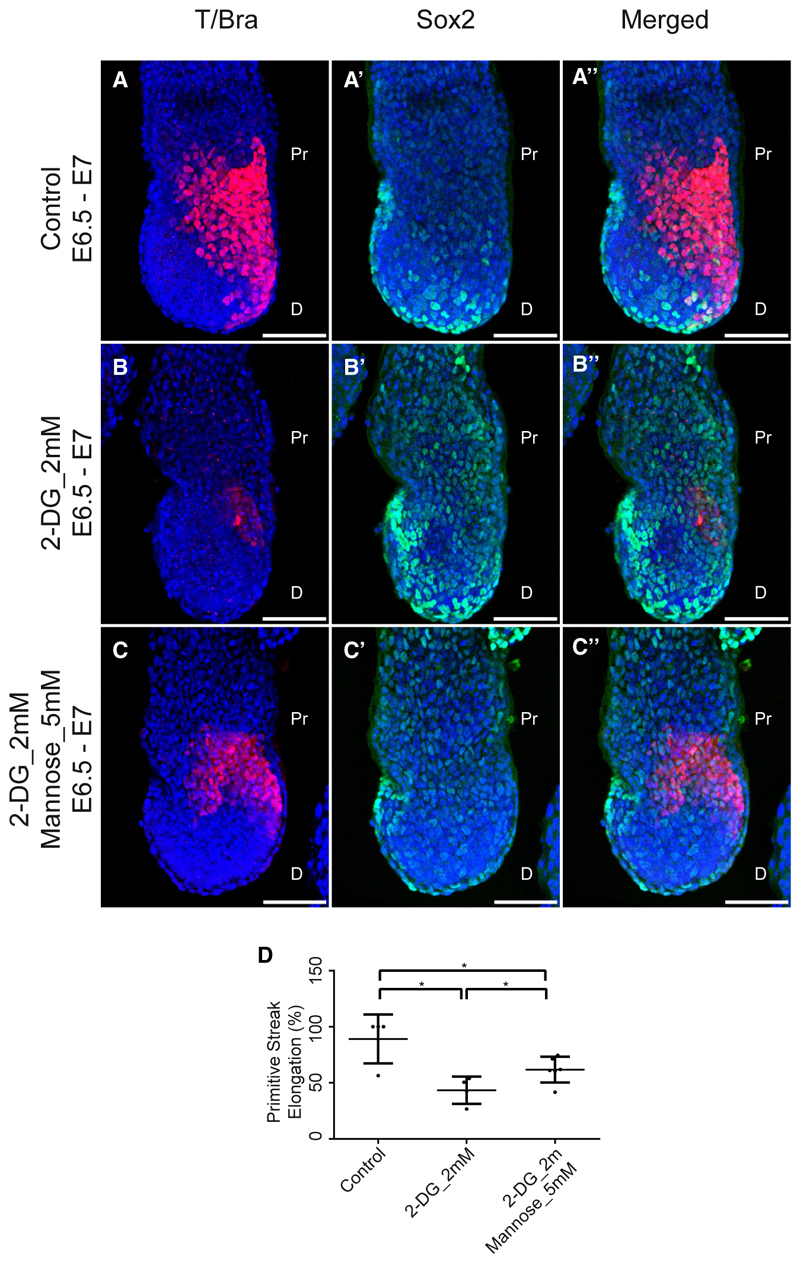
Mannose rescued 2-DG-mediated gastrulation phenotype *in vivo* mouse embryo (A–C″) Maximum intensity projections of the mouse embryos showing localization patterns of T/Bra (A–C), Sox2 (A′–B′), and both T/Bra and Sox2 merged (A″–C″) in controls (*N* = 2, *n* = 4) and in 2-DG_2 mM (*N* = 2, *n* = 4) and 2-DG_2 mM with Mannose_5 mM (*N* = 2, *n* = 6) conditions. (D) Graph showing the extent of primitive streak elongation in each condition. *p* values for control vs. 2-DG_2 mM = 0.0106, for control vs. 2-DG_2 mM Mannose_5 mM = 0.0308, and for 2-DG_2 mM vs. 2-DG_2 mM Mannose_5 mM = 0.0418. Data are represented as mean ± SEM. Scale bars, 100 μm for the confocal images (A–C″). Pr, proximal; D, distal. The blue staining denotes nuclei in all the confocal images.

Having established the role of mannose in mesoderm specification in gastruloids, we wanted to test whether it had a role to play *in vivo* during gastrulation. For this, we treated the mouse embryos with 2-DG or 2-DG supplemented with mannose at the early streak stage around E6.5 for 12 h when the primitive streak elongated to the fullest. 2-DG-treated mouse embryos showed a weak localization of T/Bra as compared with the controls while addition of mannose restored the localization (compare Figure 5B with 5A and 5C). Localization of Sox2 appeared to have increased and expanded to the posterior region as compared with the controls (Figures 5A′–5B′). This observation was in line with the previous studied in the tailbud of the chicken embryo where 2-DG treatment caused the expansion of Sox2-positive cells at the expense of the T/Bra-positive cells.^8^ Addition of mannose to 2-DG-treated mouse embryos led to a reduction reduced Sox2 levels back to that of the control embryo (Figure 5C′). The rescue in *T/Bra* expression is matched by a rescue in primitive streak elongation (Figure 5D).^26^ These results demonstrated that mannose addition could rescue the impact of 2-DG on mesoderm specification and primitive streak elongation during mouse gastrulation.

### Depriving intracellular mannose affected mesoderm specification

To assess whether mannose is required for mesoderm specification in gastruloids, we next aimed to deplete its intracellular levels. Mannose is an epimer of glucose where glucose-6-phosphate gets converted to fructose-6-phosphate and subsequently to mannose-6-phosphate catalyzed by phosphomannose isomerase (PMI) in a reversible reaction. In addition to *de novo* synthesis, mannose can also be recycled from existing glycoproteins through multiple routes. First, misfolded glycoproteins are stripped of their glycans, and mannose is released back into the pool to be utilized again. Second, glycans of correctly folded glycoproteins are extensively processed in the ER and the Golgi releasing mannose. Third, endocytosed glycoproteins are degraded in lysosomes, releasing mannose.^27^ All these reactions are carried out by compartment-specific mannosidases.^28^ In this experiment, we aimed at inhibiting both these pathways of mannose production between days 3–4 similar to the 2-DG treatment using specific chemical inhibitors and evaluating its effect on mesoderm specification.

**Figure 6:**
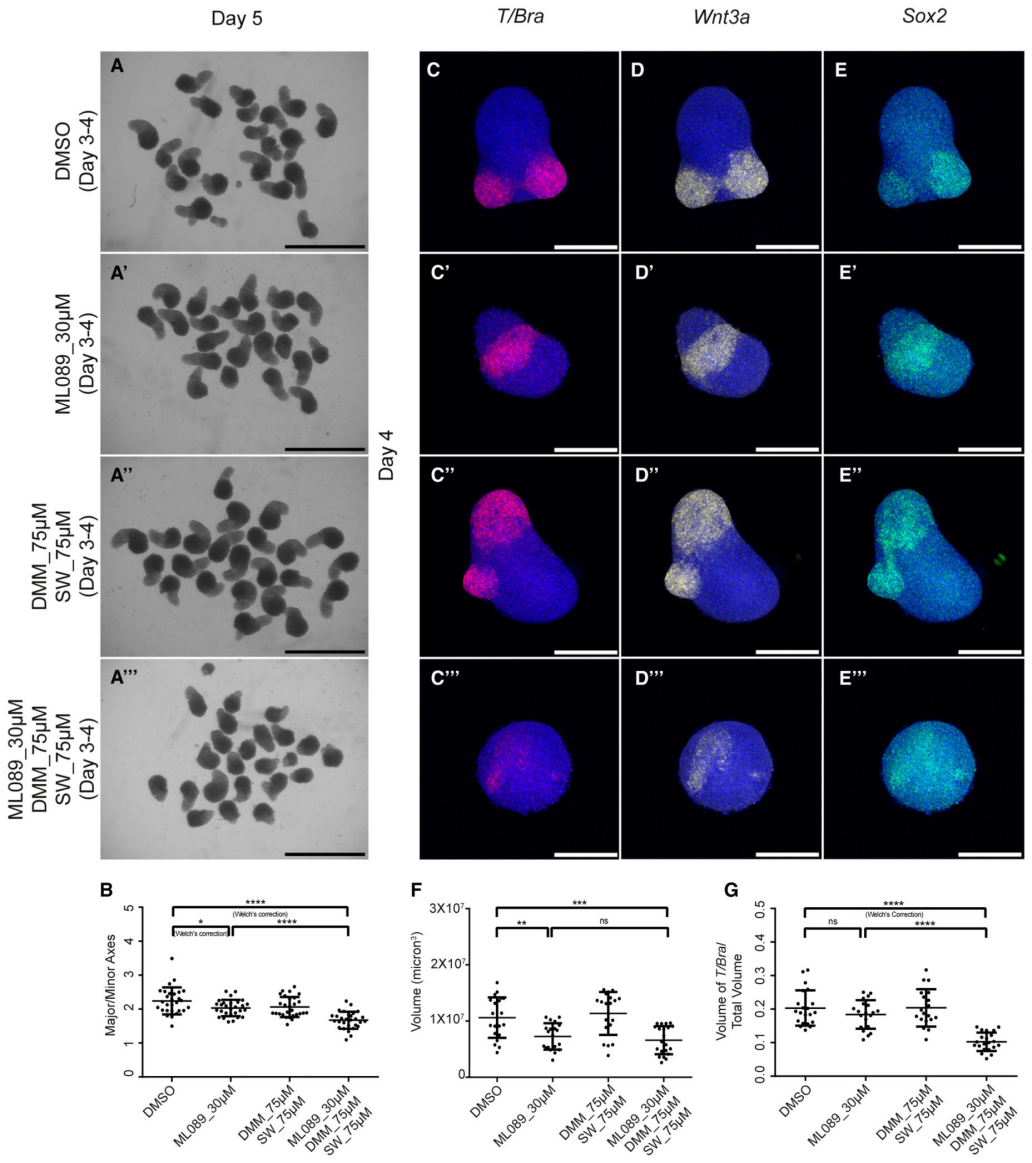
Depriving intracellular mannose affected mesoderm specification (A–A‴) Overview images showing day 5 gastruloids treated with DMSO (A), ML089_30 μM (A′), DMM_75 μM SW_75 μM (A″), and ML089_30 μM DMM_75 μM SW_75 μM (A‴) (*N* = 3). (B) Graph showing the ratio of the major axis to minor axis of the gastruloids calculated from (A–A‴) images. (*N* = 2, DMSO = 28, ML089_30 μM = 28, DMM_75 μM SW_75 μM = 28, and ML089_30 μM DMM_75 μM SW_75 μM = 29.) *p* values for DMSO vs. ML089_30 μM = 0.0211, for DMSO vs. ML089_30 μM DMM_75 μM SW_75 μM < 0.0001, and for ML089_30 μM vs. ML089_30 μM DMM_75 μM SW_75 μM < 0.0001. Data are represented as mean ± SEM. (C–E‴) Maximum intensity projections of the HCR staining in day 4 gastruloids showing the expression patterns of *T/Bra* (C–C‴), *Wnt3a* (D–D‴), and *Sox2* (E–E‴) in DMSO (C, D, and E: *N* = 2, *n* = 21); ML089_30 μM (C′, D′, and E′: *N* = 2, *n* = 20); DMM_75 μM SW_75 μM (C″, D”, and E″: *N* = 2, *n* = 19); and ML089_30 μM DMM_75 μM SW_75 μM (C‴, D‴, and E‴: *N* = 2, *n* = 21) conditions. (L) Graph showing the total volume of day 4 gastruloids in each condition (*N* = 2; DMSO, *n* = 21; ML089_30 μM, *n* = 20; DMM_75 μM SW_75 μM, *n* = 19; and ML089_30 μM DMM_75 μM SW_75 μM, *n* = 21.) *p* values for DMSO vs. ML089_30 μM = 0.0011, for DMSO vs. ML089_30 μM DMM_75 μM SW_75 μM = 0.0001, and for ML089_30 μM vs. ML089_30 μM DMM_75 μM SW_75 μM = 0.3886. Data are represented as mean ± SEM. (M) Graph showing the volume of *T/Bra* expression domain normalized to the total volume of day 4 gastruloids in each condition (*N* = 2; DMSO, *n* = 21; ML089_30 μM, *n* = 20; DMM_75 μM SW_75 μM, *n* = 19; and ML089_30 μM DMM_75 μM SW_75 μM, *n* = 21.) *p* values for DMSO vs. ML089_30 μM = 0.2052, for DMSO vs. ML089_30 μM DMM_75 μM SW_75 μM < 0.0001, and for ML089_30 μM vs. ML089_30 μM DMM_75 μM SW_75 μM < 0.0001. Data are represented as mean ± SEM. Scale bars, 2 mm for the overview images (A–A‴) and 200 μm for the confocal images (C–E‴). The blue staining denotes nuclei in all the confocal images. DMM, 1-deoxymannojirimycin; SW, swainsonine.

We first blocked *de novo* synthesis using the chemical inhibitor ML089, which is an orally available drug developed to manage congenital disorders of glycosylation 1a (CDG1a).^29^ ML089 alone had a mild effect on the axial elongation as revealed by the aspect ratio measurement of day 5 gastruloids (Figures 6A, 6A′, and 6B). HCR analysis for *T/Bra, Sox2*, and *Wnt3a* in day 4 gastruloids showed no changes in their expression levels (Figures 6C–6E and 6C′–E′). ML089-treated gastruloids, though, were smaller, but the expression domain of *T/Bra* was scaled accordingly on day 4 (Figures 6F and 6G). We next blocked the mannose-recycling pathways using swainsonine (SW), an alkaloid, and 1-Deoxymannojirimycin (DMM), a mannose analog that inhibited the lysosomal and Golgi-specific mannosidases.^30,31^ Alone, this treatment did not have any effect on the axial elongation on day 5 as well as the expressions of *T/Bra, Sox2*, and *Wnt3a* (Figure 6B and compare Figure 6A″ with 6A, and 6C–6E with 6C″–6E″). It did not have any effect on the size of gastruloids as well as the *T/Bra* expression domain (Figures 6F and 6G). However, blocking the *de novo* synthesis and the recycling pathways together led to a significantly shorter axis and weaker *T/Bra* and *Wnt3a* expressions as compared with controls (Figure 6B and compare 6A‴ with 6A, 6C‴ with 6C, and 6D‴ with 6D). The expression domain of *T/bra* was not scaled according to the size of the gastruloids (Figures 6F and 6G). Altogether these results showed how a fine balance of mannose level was achieved by integrating inputs from the *de novo* and recycling pathways to bring about proper mesoderm specification and axial elongation in gastruloids.

### Proteomics analysis revealed 2-DG affecting glycosylation of a Wnt pathway regulator

**Figure 7:**
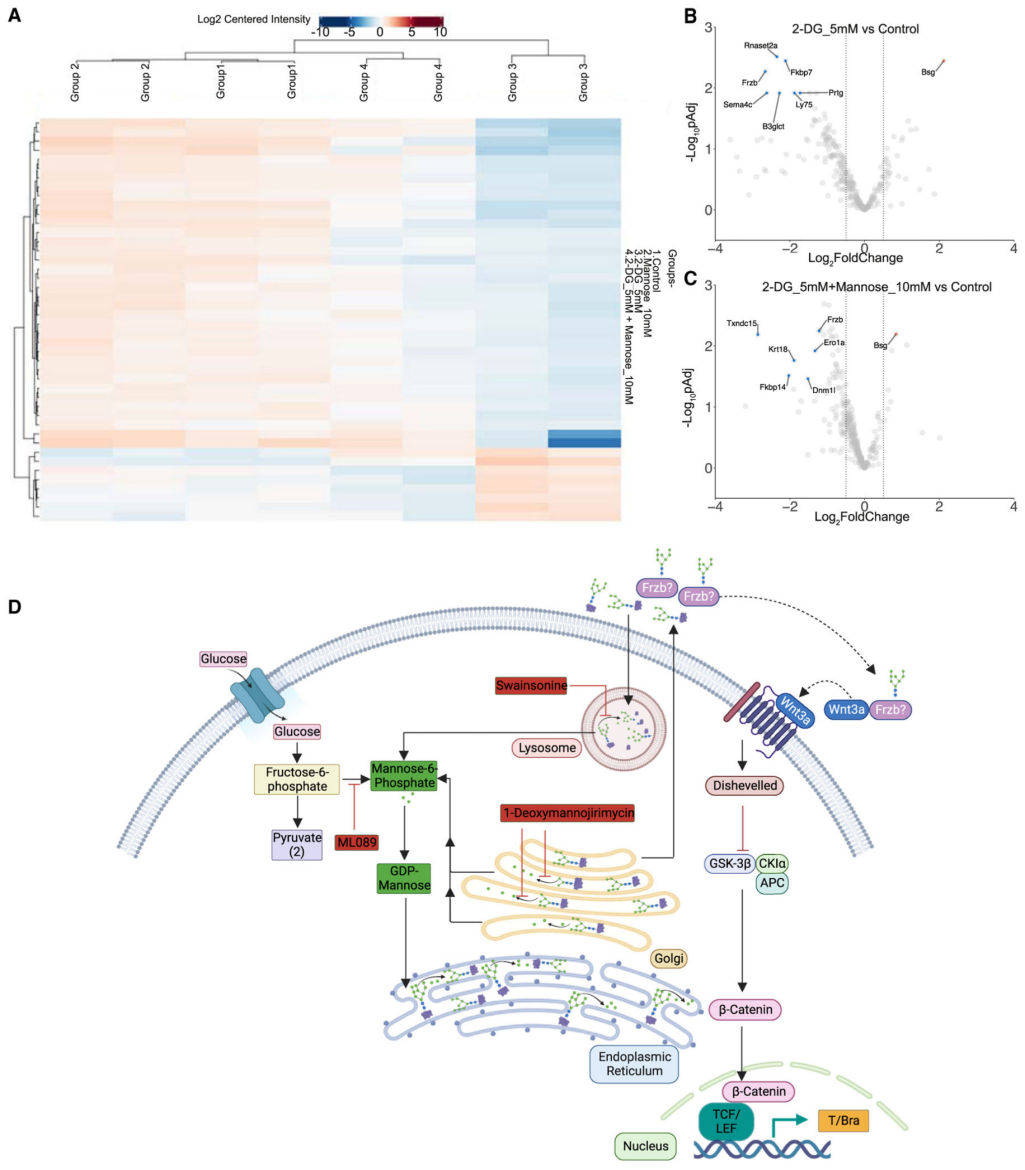
Proteomics analysis revealed 2-DG affecting glycosylation of a Wnt pathway regulator (A) Heatmap showing the abundance of total glycoproteins isolated and identified in each condition. (B and C) Volcano plot showing individual glycoproteins in lower (blue dots) and higher abundance (orange dots) in 2-DG_5 mM (B) and 2-DG_5 mM Mannose_10 mM (C) compared with control. (cut off for Log_2_FoldChange = −0.5, 0.5). (D) Schematic showing the working model of how mannose is crucial for mesoderm specification. Red boxes denote the inhibitors that block the production or recycling of mannose.

To better understand the downstream mechanisms by which mannose could rescue 2-DG treatments in gastruloids, we separated N-Glycosylated glycoproteins using Concanavilin A beads from the total protein content and performed unbiased proteomics.^32^ We hypothesized that improper glycosylation would lead to a lower recovery of glycoproteins and hence lower detection signal in the proteomics analysis. As depicted in the heatmap, there was indeed a global depletion of glycoproteins in 2-DG-treated samples as compared with the controls, mannose, and 2-DG- and mannose-treated gastruloids. Abundance-based hierarchical clustering further revealed a separation of the 2-DG sample from other clusters containing control, mannose, and 2-DG + mannose samples. Altogether, this showed that mannose restored glycosylation globally in 2-DG-treated gastruloids (Figure 7A). Interestingly, we found one candidate, Frzb, a member of the secreted frizzled-related proteins (SFRPs) that modulate Wnt signaling.^33^ Frzb was depleted in 2-DG-treated gastruloids while addition of mannose significantly elevated its level (Figures 7B and 7C).

## Discussion

Through an interrogation of the impact of 2-DG on gastruloid development, we have discovered a role of mannose in early mesoderm specification. In addition to blocking glycolysis, 2-DG competes with mannose in N-glycosylation of proteins.^19^ We have shown that mannose was sufficient to rescue 2-DG treatment in both in gastruloids and embryos. In addition, we have shown that N-glycosylation was required for mesoderm specification through mannose deprivation or glucosamine treatment. Wnt and FGF signaling pathways are involved in mesoderm specification and constitute the proteins whose glycosylation and proper intracellular trafficking are important in mediating the pathway activity. Our proteomics experiment for glycoproteins revealed that a Wnt pathway regulator and a member of SFRPs, Frzb, was depleted in 2-DG condition and restored upon addition of mannose. Frzb has been shown to be expressed in the primitive streak of the mouse embryo.^34,35^ Recently, it has been shown how SFRPs acted as carriers of Wnt3a *in vitro* and facilitated the activation of Wnt signaling.^36^ Since Frzb was not properly glycosylated in 2-DG-treated gastruloids, it could be a reason for downregulation of Wnt3a-mediated Wnt signaling, important for mesodermal fate specification^37^ (Figure 7D). Our study provided a useful experimental system to further explore the mechanistic links between hexose metabolism, protein function, and developmental signaling pathways during germ layer patterning.

This study contributes to a recent body of work focused on exploring links between central carbon metabolism and body plan formation in vertebrate embryos.^8–11^ A previous study in the chicken embryo showed that 2-DG blocked mesoderm specification in the tailbud in a glycolysis-dependent manner, which subsequently increased lactic acid secretion and increased intracellular pH.^8,9^ While we have observed that mannose treatment is sufficient to rescue the 2-DG inhibition of mesoderm markers in the context of gastruloid symmetry breaking, we cannot rule out that regulation of Wnt signaling via modifying intracellular pH is an additional mechanistic link between glycolysis and mesoderm specification in this context. Indeed, this could be the case in gastruloids as lactic acid levels were lower in 2-DG-treated gastruloids as compared with the control (Figure 3B). Interestingly, a recent study using trunk-like structures derived from mouse embryonic cell gastruloids has highlighted the importance of modulating glycolysis to achieve the appropriate balance of neural and mesodermal tissues in this system.^38^

Our study focused on the stages when mesodermal cells were already specified from the pluripotent epiblast by following gastruloids post Wnt-agonist treatment. A recent study showed that depleting glucose or treating with 2-DG at the beginning of the gastruloid development, i.e., in the pluripotent epiblast, led to an abrogation of the mesodermal fate.^39^ An explanation for these temporal differences in the impact of glucose deprivation could be that pluripotent epiblast cells depend solely depend on the *de novo* mannose synthesis pathway. Later, cells begin to maintain its steady levels of mannose, becoming refractory to glucose depletion.

Despite not requiring glucose during the onset of gastruloid elongation, a strong expression of glucose transporters co-localized with *Sox2* and *T/Bra* expressions during these stages (Figure S1), raising the question of their function in both cell types. One hypothesis is that these cells do not require glucose in the specification process but rather in other cellular processes that occur downstream and are not captured in the gastruloid assay. During gastrulation, mesodermal cells undergo an EMT,^40–43^ which is an energy expensive process.^44,45^ Indeed, recent work in the mouse embryo has demonstrated that late-stage glycolysis becomes important only after ingression through the primitive streak.^26^ Together with this work, this reveals an emerging picture of how differential hexose metabolism can be used to drive successive stages of gastrulation through the modulation of key signaling pathways such as Wnt and FGF.

## Limitations of the study

This study raised two important questions pertaining to the role of glucose in mesoderm specification and the importance of glycosylation sites on proteins, which may have a developmental significance.

We used gastruloids as a system to probe the role of glucose metabolism and found dispensable for the mesoderm specification during the time window we were studying. However, this result was different from a previous report in cardiogenic embryoid bodies (EBs) showing the expression of *T/Bra* being glucose concentration dependent.^46^ Mesoderm specification and its time window under investigation in EBs were different from that in the gastruloids. Nonetheless, our study complemented other recent reports showing how the dependency on glucose for mesoderm specification changes as the development proceeds.^38,39^

In this study, we assayed the effect of 2-DG alone or together with mannose on glycosylation at the global proteome scale but not their effect at each of the glycosylation sites. To understand the significance of glycosylation at a particular site, further mutagenesis experiments are needed to validate its significance at a particular developmental stage in embryogenesis.

## Star⋆Methods

### Key Resources Table

**Table:** 

REAGENT or RESOURCE	SOURCE	IDENTIFIER
Antibodies		
T/bra brachyury (N-19) (For gastruloids)	Santa Cruz Biotechnology	Cat# sc-17743,RRID:AB_634980
Recombinant Anti-Glucose Transporter GLUT1 antibody [SP168]	Abcam	Cat# ab150299,RRID:AB_2920535
Glucose Transporter GLUT3 antibody	Abcam	Cat# ab41525,RRID:AB_732609
Donkey anti-Goat IgG (H+L) Cross- Adsorbed Secondary Antibody, Alexa Fluor™ 568	Thermo Fisher Scientific	Cat# A-11057,RRID:AB_2534104
Donkey anti-Rabbit IgG (H+L) Highly Cross- Adsorbed Secondary Antibody, Alexa Fluor™ 647	Thermo Fisher Scientific	Cat# A-31573,RRID:AB_2536183
Brachyury (D2Z3J) Rabbit mAb (For the mouse embryos)	Cell Signaling Technology	Cat# 81694,RRID:AB_2799983
SOX2 Monoclonal Antibody (Btjce), eBioscience (For mouse embryos)	Thermo Fisher Scientific	Cat# 14-9811-82,RRID:AB_11219471
Chemicals, peptides, and recombinant proteins		
Glasgow’s MEM (GMEM)	ThermoFisher Scientific	Cat# 11710035
DMEM/F-12, no glutamine	ThermoFisher Scientific	Cat# 21331020
Neurobasal™ Medium	ThermoFisher Scientific	Cat# 21103049
Embryonic Stem Cell FBS, Qualified, One Shot™ Format, US Origin	ThermoFisher Scientific	Cat# 16141002
ESGRO® Recombinant Mouse LIF Protein	Sigma-Aldrich/Merck	Cat# ESG1106
2-Mercaptoethanol	ThermoFisher Scientific	Cat# 31350010
MEM Non-Essential Amino Acids Solution (100X)	ThermoFisher Scientific	Cat# 11140050
Sodium Pyruvate (100 mM)	ThermoFisher Scientific	Cat# 11360070
L-Glutamine (200 mM)	ThermoFisher Scientific	Cat# 25030149
Dimethyl sulfoxide	Sigma-Aldrich/Merck	Cat# D2438
CHIR99021	Sigma-Aldrich/Merck	Cat# SML1046
PD 0325901	Sigma-Aldrich/Merck	Cat# PZ0162-5MG
B-27™ Supplement (50X), serum free	ThermoFisher Scientific	Cat# 17504044
2-Deoxy-D-glucose	Sigma-Aldrich/Merck	Cat# D8375
Phosphate buffered saline	Sigma-Aldrich/Merck	Cat# P4474
ML089	MCE (MedChemExpress)	Cat# HY-138802
Swainsonine	Sigma-Aldrich/Merck	Cat# S8195-1MG
1-Deoxymannojirimycin	Sigma-Aldrich/Merck	Cat# D9160-5MG
DMEM/F12 without glucose	Biowest	Cat# L0091-500
Neurobasal™-A Medium, no D-glucose, no sodium pyruvate	ThermoFisher Scientific	Cat# A2477501
Glucose Solution	ThermoFisher Scientific	Cat# A2494001
D-(+)-Mannose	Sigma-Aldrich/Merck	Cat# M6020
D-(+)-Glucosamine hydrochloride	Sigma-Aldrich/Merck	Cat# G1514-100G
Paraformaldehyde	Sigma-Aldrich/Merck	Cat# 158127
TWEEN® 20	Sigma-Aldrich/Merck	Cat# 655204
DAPI (4’,6-Diamidino-2-Phenylindole, Dihydrochloride)	ThermoFisher Scientific	Cat# D1306
Trypsin-EDTA (0.05%), phenol red	ThermoFisher Scientific	Cat# 25300054
TRIzol™ Reagent	ThermoFisher Scientific	Cat# 15596026
SPRISelect magnetic beads	Beckman Coulter	Cat# B23317
M-Per lysis buffer	ThermoFisher Scientific	Cat# 78503
Stable isotope labelled amino acids	Cambridge Isotope Laboratories	Cat# MSK-MET1-1
Nonidet™ P 40 Substitute	Sigma-Aldrich/Merck	Cat# 74385
Critical commercial assays		
Hybridization Chain Reaction Probes and Hairpins Version 3	Molecular Instruments	N/A
QuBit RNA High Sensitivity kit	ThermoFisher Scientific	Cat# Q32852
NEBnext Poly (A) magnetic isolation module	NEB (New England Biolabs)	Cat# E7490S
NEBNext Ultra II Directional RNA library Prep Kit for Illumina	NEB (New England Biolabs)	Cat# E7760S
NEBNex multiplex oligos for Illumina Set 1	NEB (New England Biolabs)	Cat# E7335S
NEBNex multiplex oligos for Illumina Set 2	NEB (New England Biolabs)	Cat# E7500S
High Sensitivity DNA reagents kit and cassettes	Agilent	Cat# 5067-4626
PierceTM, Glycoprotein Isolation Kit Con A	ThermoFisher Scientific	Cat# 89804
Superscript III first-strand cDNA synthesis kit	ThermoFisher Scientific	Cat# 18080051
Deposited data		
Bulk transcriptomics data	This paper	GEO: GSE236887
Glycoprotein Proteomics data	This paper	ProteomeXchange: PXD048505 https://doi.org/10.25345/C5S756W60
Metabolomics data	This paper	MetabolomicsWorkbench: Datatrack_ ID:4671 Study_ID:ST003120 https://doi.org/10.21228/M86T69
Experimental models: Cell lines		
ES-E14TG2a - Embryonic stem cell with a species of origin Mus musculus	ATCC	Cat# CRL-1821,RRID:CVCL_9108
Experimental models: Organisms/strains		
Laboratory mouse CD-1	Charles River	Strain Code - 022RRID:MGI:5649524
Oligonucleotides		
See Table S1 for qPCR primers	This paper	N/A
Software and algorithms		
Fiji (ImageJ)	Schneider et al.^59^	https://imagej.nih.gov/ij/
Imaris	N/A	Oxford Instruments
GraphPad Prism	N/A	GraphPad by Dotmatics
Galaxy	N/A	https://usegalaxy.org/published/page?id=33b2b5739e9175b1
HISAT2	Kim et al.^50^	http://daehwankimlab.github.io/hisat2/
StringTie	Pertea et al.^54^	https://ccb.jhu.edu/software/stringtie/
DESeq2	Love et al.^55^	https://bioconductor.org/packages/release/bioc/html/DESeq2.html
DIA-NN	Demichev et al.^47^	https://github.com/vdemichev/DiaNN
MS-DIAL	Tsugawa et al.^49^	http://prime.psc.riken.jp/compms/msdial/main.html
R-ggplot2	Wickham et al.^60^	https://ggplot2.tidyverse.org
**D**atabase for **A**nnotation, **V**isualization and **I**ntegrated **D**iscovery	Huang et al.^51^ and Sherman et al.^58^	https://david.ncifcrf.gov/tools.jsp
Biorender	N/A	https://www.biorender.com
Other		
Zeiss LSM 700 Point Scanning Confocal Microscope (Plan-Apochromat 20X objective)	Zeiss	RRID:SCR_017377

### Resource Availability

#### Lead Contact

Further information and request for resources and reagents should be directed to and will be fulfilled by the lead contact Benjamin Steventon (bjs57@cam.ac.uk)

#### Materials Availability

No unique reagents were generated in this study.

### Experimental Model and Study Participant Details

#### Cell line

E14TG2a mouse embryonic stem cells were bought from ATCC (CRL-1821, ATCC) and cultured on the mouse embryonic fibroblasts (MEFs) for the first 3 passages. Later, the cells were weaned off the MEFs and cultured again for 2-3 passages before cry-preserving them. For these initial 5-6 passages, cells were cultured in the GMEM (11710035, ThermoFisher Scientific) along with 10% ES grade foetal bovine serum (FBS) (16141002, ThermoFisher Scientific), 1000 units/ml Leukaemia Inhibitory Factor (LIF) (ESG1106, Merck), 100 μM Beta-mercaptoethanol (31350010, ThermoFisher Scientific), 1X Non-Essential amino acids (11140050, ThermoFisher Scientific), 1mM of Sodium Pyruvate (11360070, ThermoFisher Scientific) and 2mM L-Glutamine (25030149, ThermoFisher Scientific). At this stage, cells were frozen in this medium supplemented with 10% DMSO (D2438, Merck). To plate gastruloids, frozen cells were thawed and cultured for 3 passages in 2iLIF medium which contained a Wnt agonist, CHIR99021 (3μM, SML1046, Merck), ERK antagonist PD 0325901 (1μM, PZ0162-5MG, Merck) and 1000 units/ml LIF diluted in N2B27. N2B27 medium was made using the N2 (0.5X) and B27 (0.5X) (17504044, ThermoFisher Scientific) supplements in 1:1 DMEM/F12 (21331020, ThermoFisher Scientific) to Neurobasal media (21103049, ThermoFisher Scientific). N2 supplement was prepared in house as described previously.^57^ Cells were routinely checked for mycoplasma.

#### Mouse Embryos

CD-1 mouse strain (Strain code – 022, Charles River) was used in this study. All the experiments involving the mouse embryos were performed according to the guidelines approved by the Institutional Animal Care and Use Committee at Yale School of Medicine (IACUC Approval Number – 2023-20352). The sex of the embryos was not determined during the experimentation.

### Method Details

#### Culturing Gastruloids in different glucose concentrations, inhibitor, and mannose treatments

200-300 ES cells per gastruloid were plated and cultured as previously described by Baillie-Johnson et al.^52^ Briefly, cells were trypsinised with 1 ml of 0.05% trypsin for 30 seconds to 1 min and trypsin was neutralised using 5ml of neutralisation medium (10% ES quality grade serum in GMEM). Cells were harvested by centrifuging for 5 mins at 1000 rpm at room temperature. Cells were washed twice with 5 ml of PBS containing Magnesium and Calcium to get rid of the serum traces. Cells were resuspended in 1 ml of N2B27 and counted using the Neugebauer chamber. Required number of cells was resuspended in the appropriate volume of N2B27 and 40 μl of the suspension was plated in each well of the 96 well plates. On day 2 of the protocol, each gastruloid was treated with 150ul of N2B27 containing 3μM of CHIR99091 to induce mesodermal fate. Each day after the treatment, 150 μl old medium was replaced with fresh 150 μl of N2B27 until day 5 when they elongated. Before starting with the procedure, all the reagents are pre-warmed at 37 degrees.

For the 2-DG (D8375, Merck) treatment, 500mM stock solution was made fresh in PBS (P4474, Merck) and filter sterilised using a 0.22 um filter. Gastruloids were treated between day 3 and 4 or day 4 and 5 with 5mM 2-DG diluted in N2B27. Stocks for ML089 (HY-138802, MCE MedChemExpress), Swainsonine (S8195-1MG, Merck) and 1-Deoxymannojirimycin (D9160-5MG, Merck) were made in DMSO (D2438, Merck) and single time use of 20 μl aliquots were stored at -20. The final DMSO concentration was limited to 0.5% during the treatment. For differential glucose concentration treatments between day 2 and 3 and between day 3 and 4, N2B27 was made using DMEM/F12 without glucose (L0091-500, Biowest) and Neurobasal without glucose (A2477501, ThermoFisher). For 1X and 3X glucose concentrations, 1.1 M glucose stock solution was used (A2494001, ThermoFisher Scientific) to obtain the final concentrations of 21.25mM and 63.75mM respectively. Final pyruvate concentration was adjusted to 0.36mM by adding sodium pyruvate. For the mannose (M6020, Merck) and glucosamine (G1514-100G, Merck) treatment, 500mM stock was prepared in PBS and filter sterilised. 5, 10 and 15mM working solutions of mannose were made in N2B27 with or without 5mM 2-DG. These treatments were performed on gastruloids grown in the same plate. One plate was kept as a control until day 5 to ensure that control gastruloids in the same plate elongated properly, otherwise the entire plate was discarded.

#### Culturing and treating mouse embryos with 2-DG and mannose

For the *in vivo* mouse embryo experiments, culturing and inhibitor treatments were performed according to the methods described by Cao et al.^26^ Briefly, CD-1 mice were mated, and the embryos were collected at E6.5. For 2-DG and Mannose treatments, embryos were cultured in DMEM/F12, rat serum, 1X Glutamax, 0.2X Penicillin-Streptomycin, 0.2X non-essential amino acids, at 37 degrees with 20% O_2_ and 5% CO_2_.

#### Fixing, HCR staining, immunostaining and imaging of the gastruloids and embryos

Day 4 and day 5 gastruloids were harvested from 96 well plates, washed in PBS and fixed overnight in 4% paraformaldehyde (158127, Merck). The next day, gastruloids were dehydrated in a series of increasing methanol concentrations and stored at -20 in 100% methanol at least for a day before using them for the HCR experiments. Briefly, the HCR protocol involved rehydrating the gastruloids with a descending series of methanol concentrations and finally PBS-T (0.1% Tween, 655204, Merck). Once hydrated, gastruloids were incubated in the pre-hybridisation (pre-hyb) buffer at 37 degrees for 30 minutes and then the pre-hyb buffer was replaced with HCR probes diluted in the pre-hyb buffer. Version 3.0 HCR Gene specific probes were designed by Molecular Instruments. Hybridisation took place through incubating the gastruloids with probe solution overnight at 37 degrees. The next day, the probe solution was replaced and gastruloids were washed 4 times with the washing buffer for 15 minutes at 37 degrees. This was followed by 3 × 5 minutes washes with 5X Sodium Chloride Sodium Citrate + 0.1% Tween-20 (5X SSC-T) solution. Gastruloids were then incubated with the Hairpin Amplification Buffer for 30 minutes. The fluorophore-tethered hairpins were briefly denatured before diluting them in the amplification buffer. Gastruloids were incubated overnight in the dark with the hairpin solution at room temperature. The next day, gastruloids were washed with 5X SSC-T and then PBS-T before staining them with DAPI overnight (D1306, ThermoFisherScientific). Finally, gastruloids were cleared for 24 hours in ScaleS4^48^ and imaged on the confocal microscope. All the HCR reagents and probes were bought from Molecular Instruments Inc, USA.

For the whole-mount antibody staining, gastruloids were fixed in 4% PFA overnight and washed with PBS-T three times on a shaker for 5 mins per wash. Later, the blocking, primary, and secondary antibody incubations and washes were performed as described previously with the exception of performing all the steps in a 2 ml tube.^52^ DAPI was added to the secondary antibody solution. After completing the washes, gastruloids were cleared with ScaleS4 as described above. The list of antibodies is provided in the key resource table.

HCR or antibody stained gastruloids were imaged by mounting them in ScaleS4 sandwiched between two coverslips on the Zeiss LSM700 with Plan-Apochromat 20X objective. Fixing and immunostainings of the embryos were performed according to the methods described by Cao et. al.^26^

#### Metabolomics analysis

Day 4 gastruloids from 2 plates were collected (approximately 45-48 gastruloids, per treatment, per biological replicate) (N, biological replicate = 4) into ice cold PBS in a clean glass petri dish, washed once and transferred to another glass petri dish on ice containing cold PBS. Gastruloids were collected, briefly centrifuged to settle them down and the PBS was completely removed. Gastruloids were snap-frozen in liquid nitrogen. To count the number of cells in each gastruloid, 5 gastruloids from each batch and each treatment, were collected, trypsinised for 1 min in 50 μl of 0.05% trypsin (25300054, ThermoFisher Scientific) and neutralised using 950 μl of warm N2B27. Cells were immediately counted using the Neubauer haemocytometer chamber. Snap-frozen gastruloids were sent to EMBL-Heidelberg, Germany, for the metabolomics analysis.

##### Reagents

LC-MS grade water, acetonitrile and methanol were obtained from Th. Geyer (Germany). High-purity ammonium acetate, ammonium hydroxide, and formic acid were purchased from Merck (Germany). Stable isotope labelled amino acids (MSK-MET1-1; Cambridge Isotope Laboratories, MA, USA) were used as internal standards for untargeted metabolomics.

##### Sample preparation

Metabolite extraction was performed by addition of 200 μL 80 % methanol (including 2 % (v/v) internal standards) and subsequent homogenization on dry ice via a bead beater (FastPrep-24; MP Biomedicals, CA, USA) at 6.0 m/s (5 × 30 s, 5 min pause time) using 1.0 mm zirconia/glass beads (Biospec Products, OK, USA). After centrifugation for 10 min at 15,000 × *g* and 4 °C with a 5415R micro-centrifuge (Eppendorf, Hamburg, Germany), supernatants were transferred and the remaining sample residues were reextracted with 200 μL acetonitrile:methanol:water (2:2:1, v/v) containing 1 % (v/v) formic acid using identical settings for homogenization and centrifugation. Corresponding supernatants of both extraction steps were combined and dried under a stream of nitrogen. Dried samples were reconstituted in 60 μL acetonitrile:methanol:water (2:2:1, v/v), vortexed for 5 min, centrifuged, and transferred to analytical glass vials. The LC-MS/MS analysis was initiated within one hour after the completion of the sample preparation.

##### LC-MS/MS analysis

LC-MS/MS analysis was performed on a Vanquish UHPLC system coupled to an Orbitrap Exploris 240 high-resolution mass spectrometer (Thermo Fisher Scientific, MA, USA) in negative ESI (electrospray ionization) mode. Chromatographic separation was carried out on an Atlantis Premier BEH Z-HILIC column (Waters, MA, USA; 2.1 mm x 100 mm, 1.7 μm) at a flow rate of 0.25 mL/min. The mobile phase consisted of water:acetonitrile (9:1, v/v; mobile phase phase A) and acetonitrile:water (9:1, v/v; mobile phase B), which were modified with a total buffer concentration of 10 mM ammonium acetate. The aqueous portion of each mobile phase was adjusted to pH 9.0 via addition of ammonium hydroxide. The following gradient (20 min total run time including re-equilibration) was applied (time [min]/%B): 0/95, 2/95, 14.5/60, 16/60, 16.5/95, 20/95. Column temperature was maintained at 40°C, the autosampler was set to 4°C and sample injection volume was 7 μL. Analytes were recorded via a full scan with a mass resolving power of 120,000 over a mass range from 60 – 900 *m/z* (scan time: 100 ms, RF lens: 70%). To obtain MS/MS fragment spectra, data-dependant acquisition was carried out (resolving power: 15,000; scan time: 22 ms; stepped collision energies [%]: 30/50/70; cycle time: 900 ms). Ion source parameters were set to the following values: spray voltage: 4100 V (positive mode) / -3500 V (negative mode), sheath gas: 30 psi, auxiliary gas: 5 psi, sweep gas: 0 psi, ion transfer tube temperature: 350°C, vaporizer temperature: 300°C. All experimental samples were measured in a randomized manner. Pooled quality control (QC) samples were prepared by mixing equal aliquots from each processed sample. Multiple QCs were injected at the beginning of the analysis in order to equilibrate the analytical system. A QC sample was analyzed after every 5^th^ experimental sample to monitor instrument performance throughout the sequence. For determination of background signals and subsequent background subtraction, an additional processed blank sample was recorded. Data was processed using MS-DIAL and raw peak intensities for relative metabolite quantification.^49^ Feature identification was based on accurate mass, isotope pattern, MS/MS fragment scoring and retention time matching to an inhouse library.

#### Transcriptomics analysis

Day 4 gastruloids from 1 plate (approximately 20-24 gastruloids, per treatment, per biological replicate) (N, biological replicate = 3) were collected in ice cold PBS, excess PBS was removed from the tube and initially 100 μl of TRIzol (15596026, ThermoFisher Scientific) was added to dissociate and homogenise the gastruloids. Later, 900 μl of TRIzol was added and stored in -80 until RNA extraction and library preparation. RNA was extracted using the TRIzol protocol and diluted in nuclease-free water. RNA concentration was measured using the QuBit RNA High Sensitivity kit (Q32852, ThermoFisher Scientific). For the library preparation, mRNA was isolated from 2.5 μg of total RNA using NEBnext Poly (A) magnetic isolation module (E7490S, NEB). The library was prepared as per the user’s manual using the NEBNext Ultra II Directional RNA library Prep Kit for Illumina (E7760S, NEB). Library indexing was done with NEBNex multiplex oligos for Illumina Set 1 (E7335S, NEB) and Set 2 (E7500S, NEB). Quality of the final library preparation was analysed on the Bioanalyzer using the High Sensitivity DNA reagents kit and cassettes (5067-4626, Agilent). For all the steps involving purification of library intermediates, SPRISelect magnetic beads (B23317, Beckman Coulter) were used. The transcriptomics experiment itself was outsourced to the Cancer Research UK, Cambridge. Reads obtained were analysed using the online tool, Galaxy. The pipeline consisted of trimming the index sequences using Trim_galore, obtaining BAM/SAM files using HISAT2,^50^ removing PCR duplicates using Picard Mark Duplicates followed by StringTie to assemble transcripts^54^ and DESeq2 for obtaining differentially expressed genes.^55^ Gene ontology analysis was performed using the online tool DAVID.^51,58^

#### Proteomics Analysis

120 day 4 gastruloids per condition per biological replicate (N, biological replicates = 2) were digested in 100 μl of M-Per lysis buffer (78503, ThermoFisher Scientific) together with 0.1% NP-40 detergent for 30 minutes shaking at room temperature to extract proteins in native condition, pre-requisite for glycoprotein isolation. Samples were centrifuged to remove the debris and the supernatant was later used for the glycoprotein extraction using a commercial kit, Pierce™, Glycoprotein Isolation Kit Con A (89804, ThermoFisher Scientific). User’s manual was followed to isolate glycoproteins. Glycoprotein was digested with trypsin using an S-trap procedure. Data acquisition was performed by PASEF-DIA. Peptides were loaded onto EvoTip Pure tips for desalting and as a trap column, before eluting over a 600SPD EvoSep one method onto a Bruker TimsTOF HT mass spectrometer. DIA data in .d mode were searched against and *in-silico* predicted spectral library created from SwissProt mouse entries using DIA-NN software.^47^ Searches were run at 1% FDR with the following filters applied post searching: q<0.01, protein Q <0.01 and min peptides per protein >1.

#### qPCR analysis of candidate genes

RNA was extracted as described above. cDNA was prepared using the Superscript III first-strand cDNA synthesis kit (18080051, ThermoFisher Scientific). For the standardisation of primers, control cDNA was diluted as 1:40, 1:80, 1:160 and 1:320 and the Ct values were calculated for each primer pair for a gene and compared with that of the internal control H2az1.^53^ Standardised primer sets were then used to calculate the gene expression change in different treatment groups. For this experiment, 1:80 cDNA dilution was used. A list of the primers used is provided in the Table S1.

### Quantification and Statistical Analysis

ImageJ was used for the confocal and brightfield image processing as well as the aspect ratio measurements.^59^ Volume quantifications of the *T/Bra* expression domain and the entire gastruloids were performed using Imaris. For this, gastruloids were imaged from both the sides and the overlapping confocal stacks were removed manually in ImageJ to calculate the complete volume of a gastruloid. For statistical analysis and graphs shown in Figures 1C, 2C–2E, 2I, 2J, 2L, 2M, 4H,5D, 6B, 6F, 6G, S3B, and S3D, GraphPad Prism was used. For volcano plots shown in Figures 1G, 2F, 2G, 3D, 3E, 3F, 7B, and 7C for the heatmaps shown in Figures 3B, 3C, 4I and S2 and the bar graph shown in Figure 1G, the R package ggplot2 was used.^60^ Schematic in Figure 7D is made using Biorender.Com.

The experiments were not randomised, and the investigators were not blinded during the study. The number of experiments (N), the number of samples analysed (n) and P value for the statistical tests are mentioned in the figure legends. Unpaired T-test was used unless stated as on the graphs in the figures.

## Supplementary Material

Supplemental Information

## Data Availability

The bulk RNA-seq data (GEO: GSE236887) have been deposited at the NCBI-GEO and are publicly available as of the date of publication. Metabolomics datasets (Datatrack_ID:4671, Study_ID:ST003120) have been submitted to National Institute Health (NIH) Common Fund’s National Metabolomics Data Repository (NMDR) website, Metabolomics Workbench and are publicly available as of the date of publication (MetabolomicsWorkbench: https://doi.org/10.21228/M86T69).^56^ All proteomic mass spectrometry data are referenced in ProteomeXchange (PXD048505) and can be accessed via MassIVE (MSV000093849) (ProteomeXchange: https://doi.org/10.25345/C5S756W60). Accession numbers and the DOIs for all these datasets are mentioned in the key resource table. Any additional information required to reanalyse the data reported in this work paper is available from the lead contact upon request.

## In brief

Dingare et al. identified a critical role for mannose-dependent glycosylation during early mesoderm specification both in mouse embryonic stem-cell-derived gastruloids and in gastrulating mouse embryos. Blocking intracellular mannose production and its usage in glycosylation inhibited mesoderm specification and symmetry breaking in gastruloids.

## Highlights

- 2-Deoxy-D-Glucose affects mesoderm specification through mannose inhibition
- *De novo* and salvage mannose synthesis pathways control mesoderm specification
- Mannose controls mesoderm specification in gastruloids and gastrulating embryos
- Mannose-dependent protein glycosylation is required for mesoderm specification
