# Supplementary material for: Mannose controls mesoderm specification and symmetry breaking in mouse gastruloids
Source: Dev Cell. Author manuscript; Available in PMC 2026 Apr 16. (PMC7619011; doi:10.1016/j.devcel.2024.03.031)
Supplement: Supplemental Information [file EMS213035-supplement-Supplemental_Information.pdf]

CellPress
OPEN ACCESS

SUPPLEMENTAL INFORMATION

Supplemental information can be found online at https://doi.org/10.1016/j.devcel.2024.03.031.
